# The shock, the coping, the resilience: smartphone application use reveals Covid-19 lockdown effects on human behaviors

**DOI:** 10.1140/epjds/s13688-023-00391-9

**Published:** 2023-06-05

**Authors:** Xiao Fan Liu, Zhen-Zhen Wang, Xiao-Ke Xu, Ye Wu, Zhidan Zhao, Huarong Deng, Ping Wang, Naipeng Chao, Yi-Hui C. Huang

**Affiliations:** 1 Web Mining Laboratory, Department of Media and Communication, City University of Hong Kong, 18 Tat Hong Avenue, Kowloon, Hong Kong SAR China; 2 School of Communication, Shenzhen University, 3688 Nanhai Avenue, 518060 Shenzhen, China; 3 Center for Computational Communication Research, Beijing Normal University, Zhuhai, China; 4 School of Engineering, Shantou University, Shantou, China; 5 OPPO Internet Advertising Technology, Shenzhen, China

**Keywords:** COVID-19, Lockdown, Human behaviors, Smartphone apps, Natural experiment

## Abstract

Human mobility restriction policies have been widely used to contain the coronavirus disease-19 (COVID-19). However, a critical question is how these policies affect individuals’ behavioral and psychological well-being during and after confinement periods. Here, we analyze China’s five most stringent city-level lockdowns in 2021, treating them as natural experiments that allow for examining behavioral changes in millions of people through smartphone application use. We made three fundamental observations. First, the use of physical and economic activity-related apps experienced a steep decline, yet apps that provide daily necessities maintained normal usage. Second, apps that fulfilled lower-level human needs, such as working, socializing, information seeking, and entertainment, saw an immediate and substantial increase in screen time. Those that satisfied higher-level needs, such as education, only attracted delayed attention. Third, human behaviors demonstrated resilience as most routines resumed after the lockdowns were lifted. Nonetheless, long-term lifestyle changes were observed, as significant numbers of people chose to continue working and learning online, becoming “digital residents.” This study also demonstrates the capability of smartphone screen time analytics in the study of human behaviors.

**Supplementary Information:**

The online version contains supplementary material available at 10.1140/epjds/s13688-023-00391-9.

## Introduction

Non-pharmaceutical interventions (NPIs) have been employed globally to curb the spread of COVID-19 since 2020. One class of NPIs targets limiting physical contacts, ranging from mild social distancing to drastic closure of public places and city-level lockdowns. These stringent policies were also the most effective at pandemic containment [1–6]. However, balancing epidemic control measures and their potential hazards to the economy and people’s well-being in the broader sense is essential to precision policymaking.

A critical aspect of mobility-restricting NPIs’ potential hazards is the impact on people’s daily behaviors and physiological and psychological well-being. An apparent consequence of mobility restrictions is that life’s regular routines become disrupted as people spend more time in their homes [7] and on the Internet [8] – more job was done outside of business hours [9], and night-time social media use increased [10]. Disrupted daily rhythms may have a profoundly negative impact on individuals’ emotional and mental health, especially that of the elderly and lower-income workers [11], even if physical activity can be restored through short-term interventions [12]. However, we identified two limits in existing research. First, most previous research focused on isolated behaviors or discrete periods of policy enforcement. There lacks a comprehensive understanding of how the policies may impact a wide range of human behaviors after implementation and over a long period. Second, most studies used self-reported behavioral logs as the data sources, such as time-use surveys, which are known to deviate from actual human behaviors [13, 14].

This paper utilizes smartphone data to examine the impact of lockdowns on 6 million people’s daily behavior during and after lockdowns. Smartphones, owing to the data’s broad coverage, high availability, and continuity, have become an important tool in human behavior research in recent years. For example, call detail records (CDRs) containing call and text records were used to study social relationships and networks [15, 16]; mobility data, constructed from smartphones’ periodic registrations to base towers, have been used to predict COVID-19 spread [17], reconstruct transmission events [18], and evaluate the efficacy of social distancing policies [19]. Whereas, our study focuses on a third type of data – the screen time of smartphone applications. We collected the screen time logs of all (over 14,000) apps covering a diverse market running on over two billion smartphones in China. Using bottom-up approaches, we grouped the apps into 50 market niches that target various user demands. We further identified 20 typical daily app usage patterns from all the devices, each corresponding to an interpretable user behavioral cluster.

In 2021, five Chinese provincial capital cities enacted a stringent lockdown policy, affecting over 45 million people. Each city government imposed a month-long full-scale lockdown until all infected cases were hospitalized, and no new cases were reported for two weeks. To quantify the impact of lockdowns on smartphone app use time and user behavioral patterns, we employ a dynamic difference-in-difference (DDID) approach. The effects of lockdown policies can be estimated by comparing differences between residents’ behaviors in the lockdown cities (the treated group) and cities with similar socio-demographics but never experienced lockdowns (the control group). The control group serves as counterfactual as if lockdowns did not happen in the treated group. With such a natural experiment setting, we address the following fundamental questions concerning China’s stringent lockdown policy: “Which daily activities were affected and by how much?”, “How did people cope with mobility restrictions?”, “Which populations were most affected?” and “What were the long-term impacts of the lockdown policies?”

## Data and methods

### Lockdown cities and timelines

Five provincial capital cities, i.e., Shijiazhuang, Harbin, Changchun, Guangzhou, and Nanjing, experienced outbreaks and enacted lockdowns at different times in 2021. For each of them, we selected one city for control based on their similarities in the geographical locations and socioeconomic status (Table 1; see Additional file 1, Sect. 1 for the detailed procedure). Note that lockdown policies were implemented and lifted through a series of policy announcements and enforcement measures that could span a few days. Therefore, we used the dates when the traffics from and to the lockdown cities substantially changed as the effective lockdown start and end dates (see Additional file 1, Sect. 2 for the detailed procedure.)

**Table 1:** Lockdown cities and timelines in 2021

Lockdown city	Start date	End date	Length (days)	Control city
Shijiazhuang	Jan 5	Jan 30	25	Jinan
Harbin	Jan 15	Feb 19	35	Tianjin
Changchun	Jan 15	Feb 9	25	Tianjin
Guangzhou	May 30	Jun 29	30	Zhuhai
Nanjing	Jul 19	Aug 23	35	Hangzhou

### Smartphone data and sample selection

We gathered the screen time of all applications in the lockdown and control cities logged by a leading smartphone brand. The observation period for each treated-control city pair starts two months before the lockdown starts and ends two months after the lockdown lifts. We consider a smartphone to be active if its total screen time exceeds seven hours per week during the observation period. Each active smartphone device is treated as an *active user*. The total number of active users in lockdown and control cities was 5.97 million, covering 8.1% of their total population.

Multiple apps can compete within the same market niche. Therefore, we categorize apps that provide similar services. We first filtered 679 *active apps* (used by more than $1/5000$ total active users) from more than 14,000 apps in the data. We then grouped these apps into 50 categories (see the grouping procedure and descriptions of the categories in Additional file 1, Sect. 3). Finally, we mapped these apps to a hierarchy of human psychological needs [20, 21] from materialistic to post-materialistic (see Additional file 1, Sect. 4 for elaboration.) Table 2 lists the app categories grouped and ordered by the hierarchy of human needs.^1^

**Table 2:** Fifty smartphone application market niches grouped by the hierarchy of human psychological needs

Theme	Categories
Mobility	Navigation, travel
Work	Online collaboration, productivity, job hunting
Commercial	Delayed delivery shopping, instant delivery shopping, online-to-offline (O2O) shopping, discount offers, car sales, payment, bookkeeping, seller backend, logistics
Information	News, web browser
Social	WeChat, social network service (SNS), dating, matchmaking, interest groups
Entertainment	Short video, long video, live show, video downloading, stimulating games, casual games, gaming supports, music, audiobooks, karaoke, novels, comics
Living	Local services, house hunting, car services, exercising, healthcare, new parents, finance, investing
Education	Under-12 (K12), under-18 (K18), adults, driving test
Tools	Translation, image editing, calendar, weather, phone tools

### Smartphone users’ behavior patterns and clusters

We define users’ daily smartphone usage pattern by their time allocation to each app category, i.e., $\boldsymbol{p}_{u,d}=[t_{u,1,d},t_{u,2,d},\ldots,t_{u,50,d}]$, where $t_{u,i,d}$ is user *u*’s time spent on an app category *i* on day *d*. These usage patterns could reflect the users’ socio-demographic features, hobbies, and lifestyles. We used the Latent Dirichlet Allocation algorithm to group the $\boldsymbol{p}_{u,d}$ of all users on all days into 20 clusters (see details in Additional file 1, Sect. 5). These clusters (ordered by size) could be interpreted as *regular users*, *binge-watchers*, *gamers*, *telecommuters*, *news audience*, *drivers*, *photoshoppers*, *spenders*, *adolescents & young adults*, *travelers*, *petty investors*, *homemakers*, *white collars*, *love hunters*, *sophisticated users*, *job hunters*, *new parents*, *new middle class*, *parents with small children*, and *home shoppers*. Each cluster’s signature apps are listed in Table S3. These clusters contain people’s typical daily behavioral patterns with and without the effect of lockdowns. A user belongs to only one cluster on a single day, based on the highest cluster probability yielded by the LDA algorithm. Users may stay in the same cluster or move to another on the next day.

### Dynamic difference-in-difference (DDID)

We employed a dynamic difference-in-difference (DDID) method [22] to estimate changes in app usage and user cluster sizes during and after the lockdowns. The 30 days before lockdown starts are chosen as the pre-treatment period. All five lockdowns lasted for approximately one month (the post-treatment period). We separated this period into two sections, i.e., the first and second fortnights (14 days), to characterize the immediate shock and delayed impacts of the lockdowns. The first and second 30 days after lockdowns ended are considered the short-term and long-term aftershock periods. We also use the 60-30 days prior to lockdowns as the placebo period.

We use the pre-treatment period as the baseline for estimating lockdown effects in other periods with the following ordinary least square regression:

1 $$\begin{aligned} Y_{i,t} =&\alpha _{0} + \alpha _{1}*Treated_{i} \\ &{}+ \alpha _{2}*Placebo_{t} + \alpha _{3}*Immed_{t} + \alpha _{4}*Delayed_{t} + \alpha _{5}*Short_{t} + \alpha _{6}*Long_{t} \\ &{}+ \beta _{1}*Treated_{i}*Placebo_{t} + \beta _{2}*Treated_{i}*Immed_{t} + \beta _{3}*Treated_{i}*Delayed_{t} \\ &{}+ \beta _{4}*Treated_{i}*Short_{t} + \beta _{5} Treated_{i}*Long_{t} + \epsilon , \end{aligned}$$

where $Y_{i,t}$ represents the app usage or user cluster sizes of city *i* at time *t*. $Treated$, $Placebo$, $Immed$, $Delayed$, $Short$, and $Long$ are dummy variables. $Treated_{i} = 1$ if city *i* experienced a lockdown. $Placebo_{t}=1$ if *t* falls in the placebo period; $Immed_{t}=1$ if *t* falls in the first fortnight into lockdown; $Delayed_{t}=1$ if *t* falls in the second fortnight into lockdown; $Short_{t}=1$ if *t* falls in the short-term aftershock period; $Long_{t}=1$ if *t* falls in the long-term aftershock period.

Note that app usage can be measured in both absolute screen time and relative usage compared to the pre-treatment period, i.e., divided by the average in the pre-treatment period in the same city. For the former measurement, coefficients $\boldsymbol{\beta}=\{\beta _{1},\beta _{2},\beta _{3},\beta _{4}, \beta _{5}\}$ are the changes in absolute screen time in each period. For the latter measurement, coefficients ***β*** are the percentages of changes relative to the pre-lockdown period. For user behavioral clusters, *Y* could represent their proportions in the city population as well as the relative change compared with the pre-treatment period. Coefficients ***β*** are the absolute and relative changes in the two cases, respectively. Furthermore, we expected not to observe any significant difference between the placebo and pre-treatment periods as we assume that the treated and control cities had parallel trends prior to the lockdowns.

We estimate the individual lockdown effects in each treated city as well as the overall effects on all five cities. When estimating the overall effect, the screen time and cluster sizes are the weighted averages based on city populations. Note that the five cities enacted lockdowns on different days. Therefore, we aligned the measurements in different cities by the relative time to the lockdown start dates so that all the treated samples receive the treatment virtually at the same time. That is to say, the lockdown start date is $t=0$.^2^ In this way, we avoid the potential pitfalls of estimating a staggered DID with multiple treatment periods [23]. The main text only reports the overall effect sizes. Results for individual cities are reported in Additional file 1, Sects. 6 and 7.

This model was estimated using the PanelOLS function in Python linearmodels package. Standard errors are clustered at the time level, i.e., cov_type=’clustered’ and cluster_time=True.

## Results

### Lockdowns’ immediate disruption to physical and economic activities

**Figure 1:**
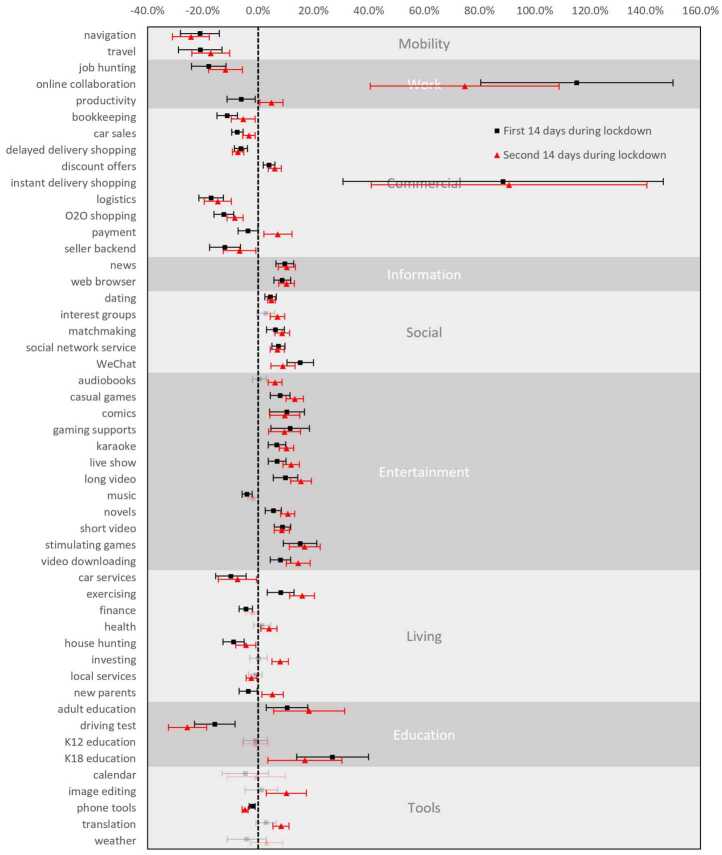
The impact of lockdowns on smartphone application screen time. Effect sizes are measured in relative screen time changes to the prior-lockdown period; error bars are 95% confidence intervals; non-significant effects are drawn using translucent hues

The five lockdowns were all characterized by the closing of public facilities, such as schools, shops, and public transportation, and the strict limitation or even prohibition of inner- and inter-city travel. Physical activities were restricted to inside homes or estates (enclosed areas with several hundreds or thousands of residents) only. Figure 1 shows the immediate impact of the lockdowns on app screen time. Naturally, mobility app usage diminished a great deal. Screen time for travel (−21.0%) and navigation (−21.1% from the period prior to the lockdowns) apps diminished immediately. The total decreased use time for navigation apps was also the largest among all apps (Fig. S5).

Delayed delivery shopping apps (−6.2%), such as Taobao, were immediately suppressed during the first fortnight as a result of restricted logistics services (−17.0%). These shopping apps also experienced the second-largest drop in absolute screen time among all the apps. Other commercial apps, such as O2O shopping (−12.4%, with which consumers locate shops and make payments), backend management for sellers (−12.0%), and car sales (−9.9%), also experienced significant decreases. Consequently, bookkeeping apps (−11.2%) were significantly less used by consumers as well. Other mobility-dependent activities, such as driving tests (−15.7%), job hunting (−17.9%), and house hunting (−8.9%), were also significantly reduced. It should be noted that impact immediacy varies among cities as lockdown policies differ slightly (see Additional file 1, Figs. 5 and 6 for details.)

Nonetheless, some commercial activities experienced a boost during the first fortnight. Notably, instant delivery shopping apps saw an 88.6% increase in use. Unlike delayed deliveries, instant deliveries are usually used to purchase daily necessities. With offline shops closed and outdoor activity restricted, people turned to instant delivery apps to stock their shelves. However, as logistic services were largely suspended during lockdowns, the feasibility of fulfilling online orders remains uncertain.

**Figure 2:**
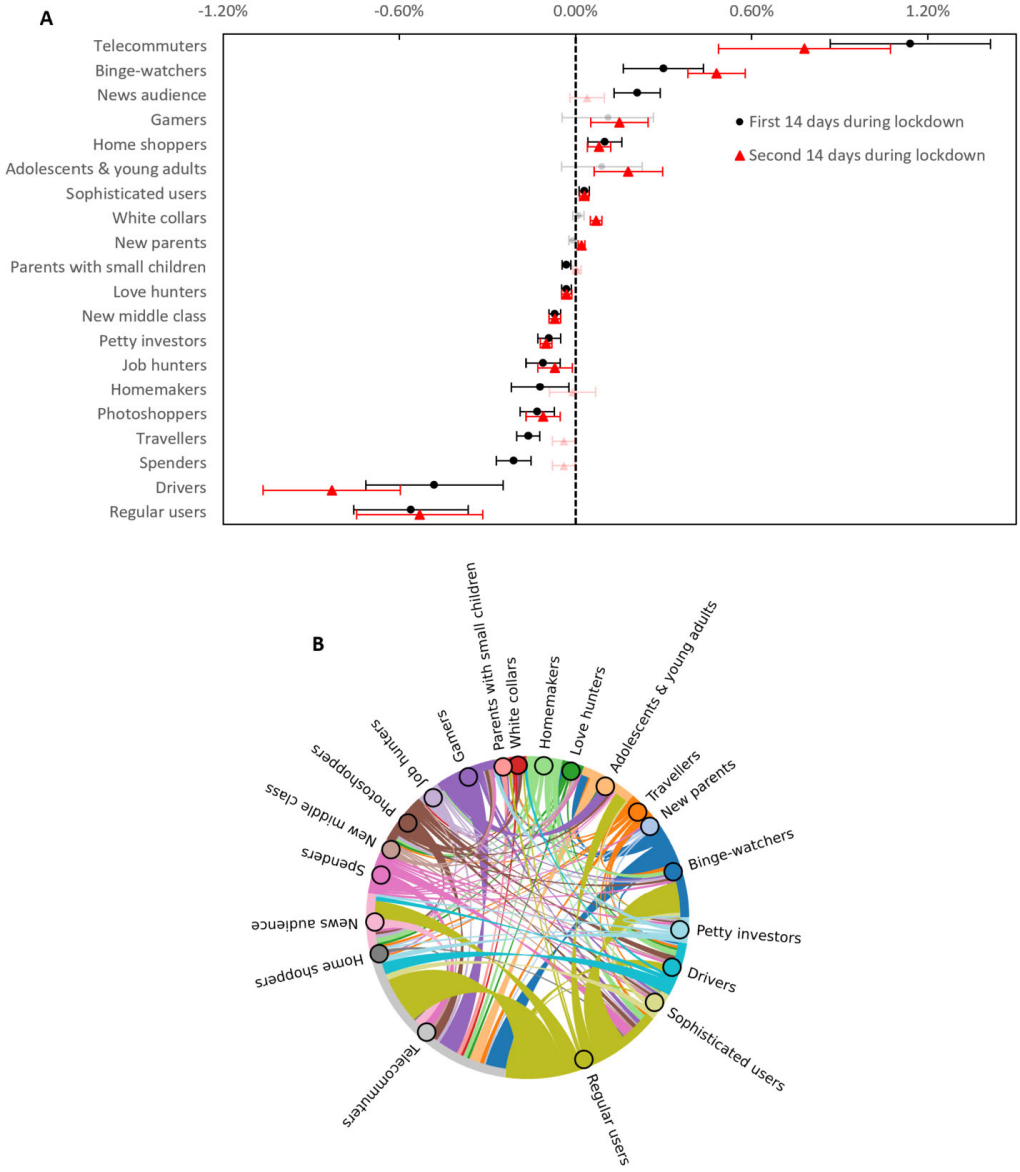
The impact of lockdowns on user behavioral patterns. - (**A**) Effect sizes are measured in the percentage of the entire population; error bars are 95% confidence intervals; non-significant effects are drawn using translucent hues. - (**B**) User behavior transitions during the first week of lockdowns. Edges are directed and originate from nodes with the same colors. Edge thickness is proportional to the total number of users that changed behaviors from one pattern to another in treated cities minus that in control cities

Lockdowns immediately disrupted smartphone users’ daily behavior patterns. *Regular users* (−0.56% of the population), *drivers* (−0.48%), *spenders* (−0.21%), and *travellers* (−0.16%) saw the most significant decreases in size (Fig. 2A). The usage pattern that saw the most significant increase in adoption was *telecommuters* (1.14%), and most new *telecommuters* were former *regular users*, *drivers*, and *gamers* (Fig. 2B).

### Natural coping during the lockdowns

As mobility was restricted, people spent more time on their smartphones. Total screen time increased by 33.0 minutes on average during the lockdowns across the five lockdown cities. While in some cities, such as Shijiazhuang, it increased for more than one hour (see Additional file 1, Fig. S2). Since physical and economic activity-related apps saw a decline, the free time filled by other smartphone apps was considerably higher than half an hour.

Lockdowns moved business and work online. Online collaboration apps saw the largest boost, recording 115.2% and 74.7% increase in the first and second lockdown fortnight, respectively. Productivity apps, such as word processing software, experienced a 6.2% drop during the first fortnight, probably due to office closings. However, their use picked up again during the second fortnight to a slight but statistically significantly higher (4.8%) level than the period prior to the lockdowns.

Sudden lockdowns induced a surge of proactive information-seeking. During the first fortnight, the use of news sites and web browsers increased by 9.6% and 8.7%, respectively. Use of these information-seeking apps increased again (to 12.9% and 11.8%, respectively) during the second fortnight.

Social activities experienced a considerable increase, with SNS apps up 7.4% and 6.9%, dating apps up 4.5% and 4.8%, and matchmaking apps up 6.2% and 8.7% during the two fortnights in the lockdowns, respectively. WeChat was the single app with the most extensive use increase.

Apps that provide entertainment services dominate the app market. As they could divert people’s attention from the pandemic and ease their pressure and anxiety [24], these apps immediately recorded higher usage and maintained the level for the remainder of the lockdowns. Apps featuring short videos increased by 8.8% and 8.6%, long videos increased by 9.9% and 15.5%, stimulating games increased by 15.2% and 16.8%, gaming support increased by 11.6% and 9.5%, comics increased by 10.4% and 9.6%, and novels increased by 5.5% and 10.7% in the two fortnights, respectively. Short video apps ranked second in the increase of absolute screen time. Notably that music apps’ usage decreased by 4% possibly due to that they are commonly used during physical commutes.

Paying more attention on health is a natural reaction to a health crisis. Apps that facilitated exercising at home saw use time increase by 8.1% and 15.9% during the two fortnights. Counter-intuitively, time spent on healthcare apps only increased during the second fortnight (3.9%), with variance observed among treatment cities (see Additional file 1, Sect. 5).

The gratification of higher-level psychological needs, e.g., self-improvement and actualization, as expressed via app use, did not always manifest an immediate increase in the lockdowns. Rather, such apps demonstrated different patterns. K18 (under 18) education apps saw a use time increase by 26.9% and 16.9% during two fortnights, i.e., an immediate burst. However, adult education apps saw use time increase by 10.4% and 18.4% in the two fortnights, respectively, showing a late increment of attention. Similarly, translation apps used for reading and learning foreign languages only saw an increase by 8.3% in the second fortnight. Nonetheless, education apps for kids did not share the increasing trend of other educational apps. Such apps are typically used by young parents in home education settings and thus contend with other activities, such as career work and housework.

Coping with lockdowns can reinforce or change daily routines. The number of users with patterns *binge watcher* (by 0.30% and 0.48% of the population in the two fortnights, respectively), *news audience* (0.21% in the first fortnight), and *gamer* (0.15% in the second fortnight) saw significant increases. *Regular users* contributed the most proportion to *binge watchers* and *news audiences*.

### Behavioral resilience

**Figure 3:**
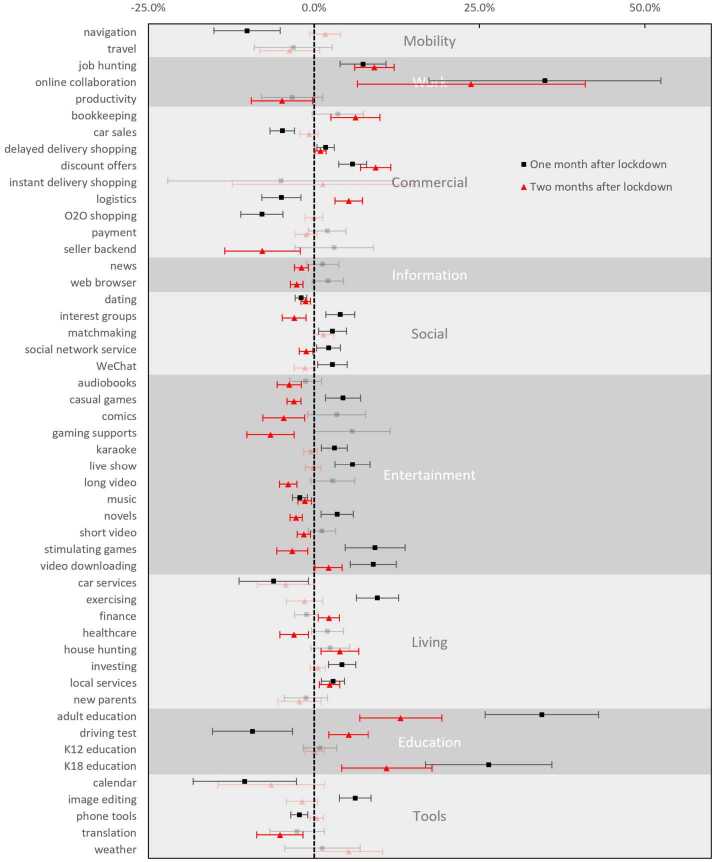
The aftershock of lockdowns on smartphone application screen time. Effect sizes are measured in relative screen time changes to the prior-lockdown period; error bars are 95% confidence intervals; non-significant effects are drawn using translucent hues

As lockdown policies lifted gradually, smartphone usage gradually returned to normal (on average 9.3 minutes more for another month and 9.4 minutes less in the second month). Most apps also resumed their pre-lockdown usage in the first or second month after the lockdowns (Fig. 3).

However, screen time for some apps remained elevated for two months after the lockdowns. Online collaboration apps retained high use rates (up 34.9% and 23.8% in the first and second months, respectively) after the lockdowns. Education apps, especially for adults (34.4% and 13.1%) and adolescent (K18) learners (26.4% and 11.0%), also maintained usage increases.

Interestingly, some apps experienced post-lockdown “retaliations” – a bounce back from suppression or a decline from incitement. For example, some commercial activities, such as discount offers (5.8% and 9.3% in the two months after the lockdowns, respectively), job hunting (7.4% and 9.1%), logistic services (5.2% in the second month), bookkeeping (6.3% in the second month), and house hunting (3.9% in the second month) logged higher usage levels. On the contrary, many entertainment activities, such as stimulating games (−3.3%), casual games (−3.1%), gaming support (−6.6%), long video (−3.9%), short video (−1.6%), comics (−4.6%), novels (−2.7%), social activities, such as social network services (−1.2%), dating (−1.3%), and interest groups (−3.0%), and information seeking activities, i.e., news (−1.9%) and web browsering (−2.6%), saw decreased use in the long run (two months after the lockdowns). These patterns reflect repulsion after restricted behaviors or fatigue after excessive behaviors for a long time.

**Figure 4:**
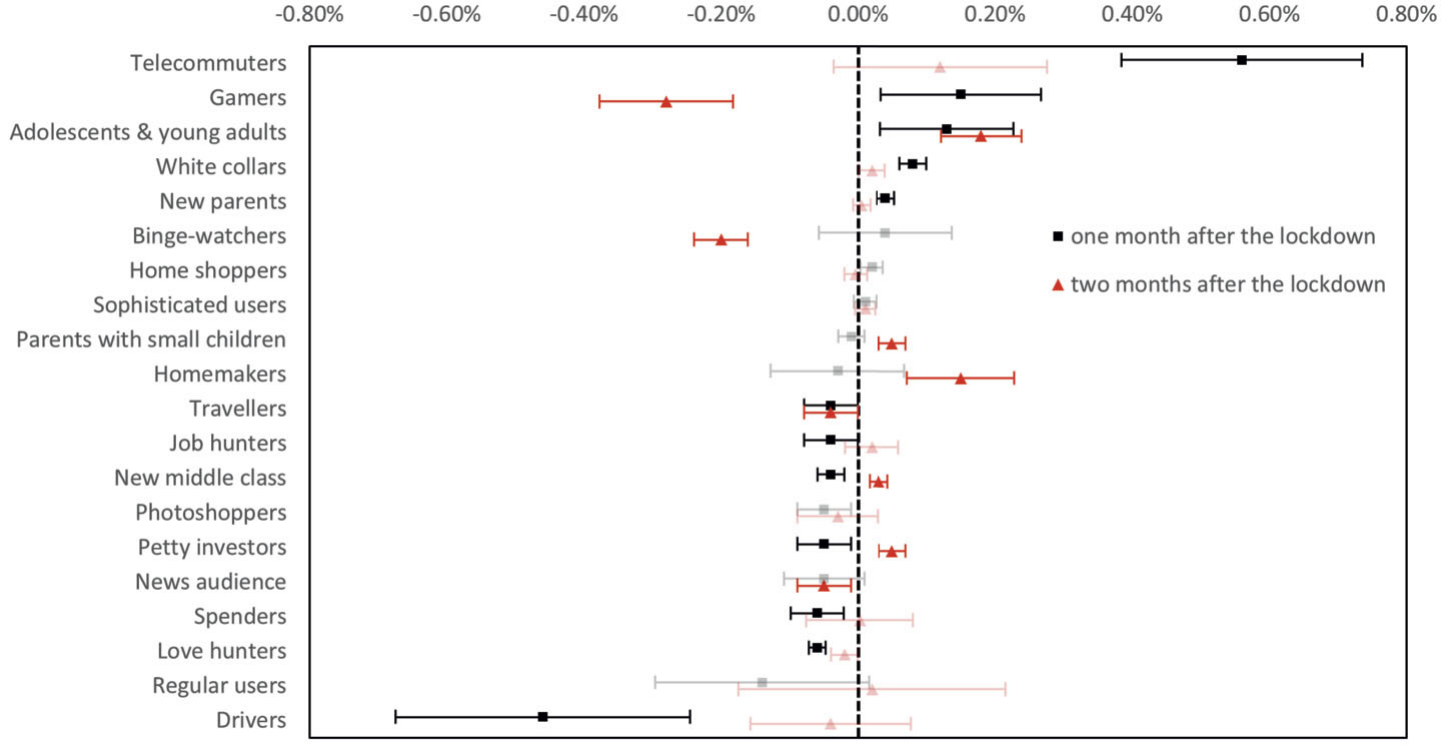
The aftershock of lockdowns on user behavioral patterns. Effect sizes are measured in the percentage of the entire population; error bars are 95% confidence intervals; non-significant effects are drawn using translucent hues

Despite of the behavioral resilience of most people (Fig. 4), a significant percentage of the entire population changed their behaviors over the long term (2.1% and 1.3% in the two months after the lockdowns, respectively). Similar to the “retaliation” effect of certain apps’ use rates, some user clusters that expanded during the lockdowns shrank to a size smaller than the baseline period prior to lockdown. In particular, *gamers*, *binge-watchers*, and *news audiences* recorded losses over the long term (−0.28%, −0.20%, −0.05% of the entire population). *Homemakers*, while decreased in number during the lockdowns, increased by 0.15% of the population two months after the lockdowns. Meanwhile, some users retained lockdown-induced behavioral changes. *Adolescents & young adults* retained their lockdown growth numbers (increased by 0.18% of the entire population) in the second month after the lockdowns. While, the number of travelers (−0.04%) struggled to recover.

## Conclusion and discussion

How COVID-19 policies affect people’s overall mental and physical well-being is an essential consideration for policy-making. Lack of empirical guidance on these questions prevents policymakers from making precise decisions during rapid developments in pandemic conditions. Our observational study, conducted using millions of smartphones, enabled us to scrutinize one particular policy—city-level lockdowns—and assess human behavioral changes before, during, and after its implementation with a high degree of accuracy.

Previous research found that, among all COVID-19 policies, lockdowns may have had the strongest negative impact on the economy [25]. We further identified the economies most affected. Online shopping and the automobile industry, which comprise a significant proportion of China’s GDP, paused for almost three months due to the closure of physical stores and logistics obstructions. Nonetheless, these businesses showed strong resilience, as they eventually resumed from the suppressed levels during lockdowns.

We also discovered that individuals tend to prioritize immediate gratification over long-term edification when making use of their newfound free time during lockdowns, as psychological theory predicted [19]. At the base of the human needs pyramid, a continuous physical demand for daily essentials prevails, even as usage of other commercial applications ceases. This observation should serve as a reminder for policymakers to prioritize ensuring a steady supply of daily necessities in order to mitigate the impact of lockdowns. In response to the staggering impact of lockdowns, depression, anxiety, and distress have emerged as prevalent emotions [26]. We have noted that the need for information, entertainment, and social interaction swiftly rose to prominence, consequently leading to a notable surge in screen time during these restrictive periods. However, such a trend can also shade into a degree of loss of self-control [27] or even lead to pathologies, such as ‘Nomophobia’ (no-mobile-phone-phobia) and gaming disorder [28, 29].

A silver lining emerged as we noticed no lasting significant surge in the utilization of social and entertainment apps, at least from a collective perspective. The majority of apps catering to lower-level needs reverted to regular or even suppressed usage after the lockdown, and most users resumed their daily routines. On the contrary, higher-level needs ultimately prevailed and persisted beyond the lockdown. The implication is apparent: humans are adaptive to a changing environment. Such resilience has asserted itself repeatedly throughout history [30] and is supported by recent literature, e.g., an increased online productivity amid the pandemic [31]. Nonetheless, behavioral adaptations in response to the pandemic may not consistently contribute to effective epidemic control. Compliance with high-cost and sensitizing measures, such as physical distancing, may diminish as individuals experience “pandemic fatigue” due to extended policy implementation [32]. We, therefore, argue that strong NPI measures, once used, must be swift, strict, and succinct. People could have enough resilience to cope with short lockdowns, but their adherence might not sustain beyond a certain threshold.

This study presents certain limitations that warrant consideration. First, this natural experiment might be unique, given that China was among the few countries that still adopted a “zero COVID” strategy in 2021. Nonetheless, we believe that behavioral changes observed in response to the lockdown policies in China can be interpreted universally. Secondly, despite a significant surge in smartphone usage, smartphone app data only captures six hours of human activity within a 24-hour period. It is plausible that lockdowns altered human behaviors for the remaining 18 hours. Traditional surveys or constantly recorded smartphone mobility data could be paired with app use to develop a more comprehensive picture. Further, one could use time series analysis to tap into the daily activity sequences, e.g., the order of and switching between apps, to explore more behavioral variables. Lastly, the smartphone manufacturer does not collect the socio-demographic information of users, which inevitably leads to the sample representativeness bias. Nevertheless, as the manufacturer holds a top-3 market share in China and sells both high-end and low-end smartphones, we believe that the users in our dataset would cover the full spectrum of demographics in the population.

## Supplementary Information

Below is the link to the electronic supplementary material.

Supplementary information (DOCX 6.7 MB)

## Data Availability

Data were collected and analyzed in Shenzhen HeyTap Technology Co., Ltd., a leading Chinese smartphone manufacturing company. Aggregated level app usage and user cluster size datasets and the computer codes used for calculating the DDID effects can be found in https://github.com/abcdefg3381/smartphone_usage. Government policy enforcement timelines (in Chinese) can also be found in the repository.

## Footnotes

1 The miscellaneous phone tools which do not map to any human need directly and are listed at the end of the table.

2 Considering that the duration of the lockdowns in different cities varied, we only used the first 25 lockdown days and discarded the rest for cities with longer lockdown duration. In this case, the second-fortnight period overlapped with the first fortnight on days 12, 13, and 14.

## Abbreviations

CDR: call detail records
DDID: dynamic difference-in-difference
DID: difference-in-difference
K12/18: under 12/18
LDA: Latent Dirichlet allocation
NPI: non-pharmaceutical intervention
O2O: online-to-offline
SNS: social network service
