# Supplementary material for: The shock, the coping, the resilience: smartphone application use reveals Covid-19 lockdown effects on human behaviors
Source: EPJ Data Sci. 2023 Jun 5;12(1):17. doi: 10.1140/epjds/s13688-023-00391-9 (PMC10240109; doi:10.1140/epjds/s13688-023-00391-9)
Supplement: Supplementary file 1 — Supplementary information (DOCX 6.7 MB) [file 13688_2023_391_MOESM1_ESM.docx]

**Supplementary Materials**

# **1. Selection of control cities**

Table S1 Lockdown and control cities’ sociodemographic features (lockdown cities in grey). The cities’ sociodemographic data were published by the National Bureau of Statistics, China, in 2019.

| **City** | **Geographical**  **location in China** | **Population**  **(M)** | **GDP**  **(B)** | **No. active users** | **Active users in the population** |
| --- | --- | --- | --- | --- | --- |
| Shijiazhuang | Central north | 10.5 | 593.5 | 829,488 | 7.9% |
| Jinan | Central north | 8.0 | 1,014.1 | 554,817 | 6.9% |
| Harbin | Northeast | 9.5 | 518.4 | 491,549 | 5.2% |
| Changchun | Northeast | 7.5 | 663.8 | 557,930 | 7.4% |
| Tianjin | Central north | 11.1 | 1,408.4 | 926,304 | 8.3% |
| Guangzhou | South | 9.5 | 2,501.9 | 1,182,381 | 12.4% |
| Zhuhai | South | 2.4 | 348.2 | 208,222 | 8.7% |
| Nanjing | East | 7.1 | 1,481.8 | 489,283 | 6.9% |
| Hangzhou | East | 8.0 | 1,610.6 | 734,903 | 9.2% |

Ideal control cities were (1) geographically approximate to lockdown cities but not directly adjunct to control for spillover effects (Holtz et al., 2020); and (2) had comparable population and gross domestic product (GDP). **Table** S1 shows the control cities that we chose for each lockdown city. Shijiazhuang-Jinan and Nanjing-Hangzhou are ideal treatment-control pairs as their geographical location, population, GDP, and the number of HeyTap users were very similar. The ideal control city for Harbin and Changchun is Shenyang, another provincial capital city geographically located between the two, and the ideal control for Guangzhou is Shenzhen, the only other southern Chinese city with around 10 million people. However, Shenyang and Shenzhen also experienced small-scale outbreaks in 2021, and their residents’ smartphone use behavior changed slightly. We, therefore, selected Tianjin, a major northern Chinese city, as the control for Harbin and Changchun instead. Zhuhai, a quite economically developed city in the same Province as Guangzhou, served as the control for Guangzhou.

# **2. Lockdown timeline**

The lockdowns were implemented and lifted through a series of policy announcements and enforcement measures that spanned a few days. This practice further varied among cities. As a result, we determined lockdown start dates by observing when lockdowns began to affect people’s mobility. We first collected the inbound and outbound traffic indices to and from all the treated and control cities with the Baidu Maps service^[[1]](#footnote-1)^. We then calculated the ratio of total traffic (inbound and outbound) of each treated city to the corresponding control city. Finally, we used a change-point detection algorithm, i.e., the linearly penalized segmentation model with a Gaussian kernel (Killick, Fearnhead, & Eckley, 2012), on the ratios to determine the lockdown dates. We chose the change points that fall into the consecutive lockdown policy start and end dates as the effective lockdown start and end dates, respectively (see **Fig.** S1). The lockdown policies were collected from national news and local health authorities’ social media accounts. We have tested lockdown dates with other possible definitions, and the results are robust.

The average total smartphone use time in the nine cities is plotted in **Fig.** S2. Cities that experienced lockdowns and their control peers showed parallel trends before the lockdowns, demonstrating the validity of dynamic difference-in-difference (DDID) modeling.


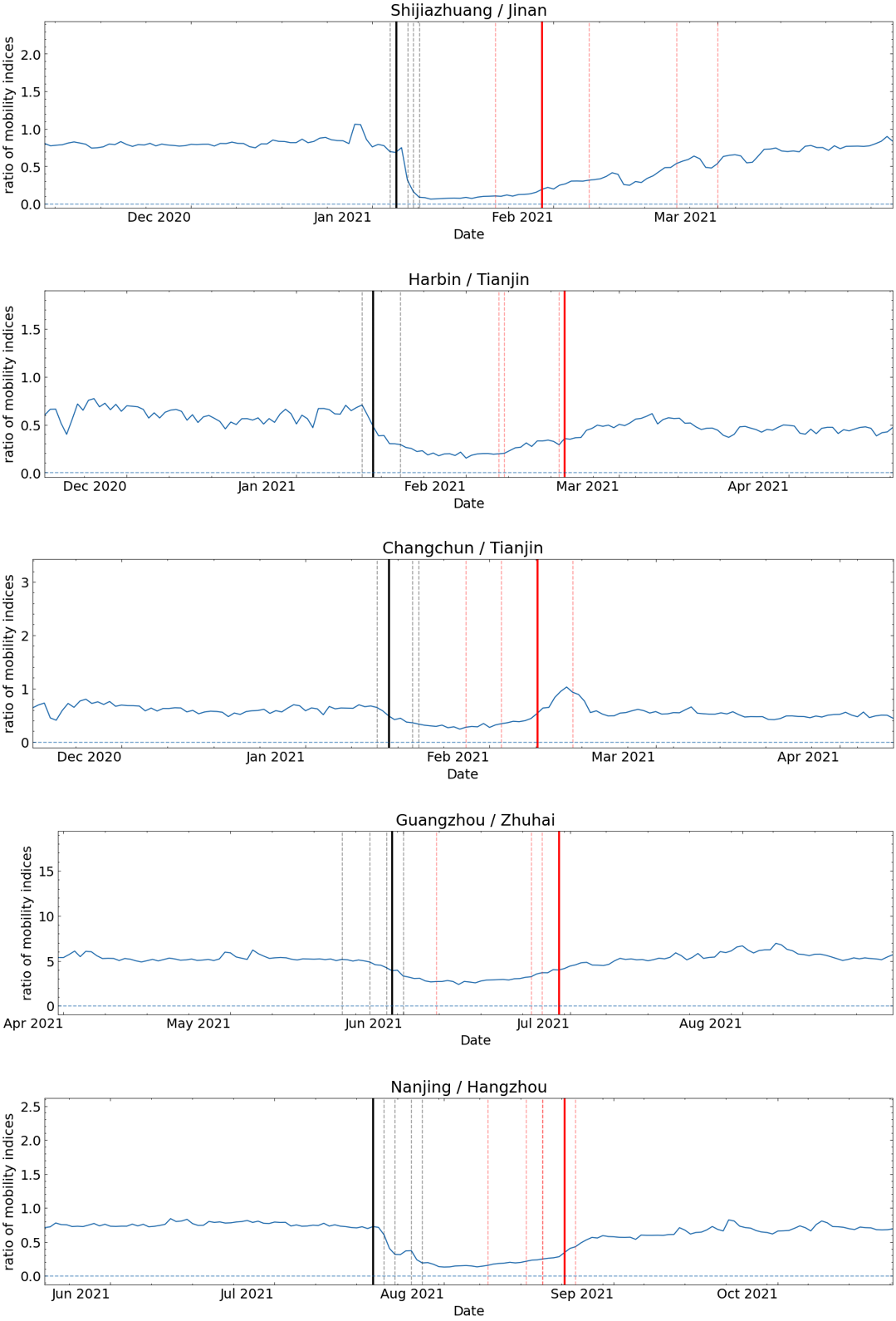


Figure S1 Ratio of mobility index in the lockdown city to the control city. The solid vertical lines indicate change points in the time series. Vertical dashed lines indicate the announcement dates for major lockdown start (black) and end (red) policies.


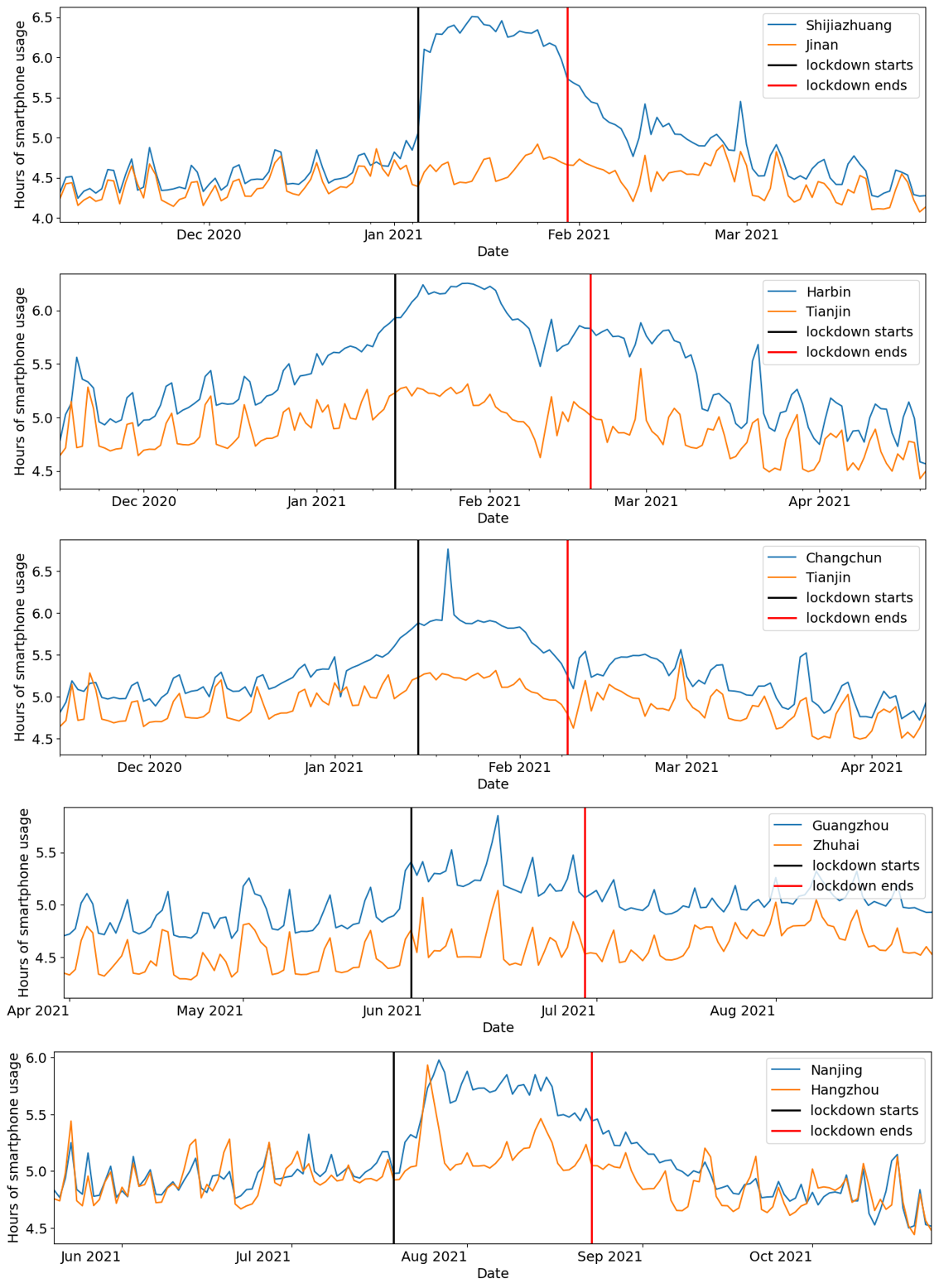


Figure S2 Total smartphone usage hours in lockdown and control cities. Vertical lines indicate lockdown start and end dates. Total smartphone usage increased during lockdowns in all treated cities and disrupted periodic (weekly) patterns in some, e.g., Shijiazhuang, Harbin, and Changchun. In Guangzhou and Nanjing, periodic patterns were preserved. Slightly varied lockdown policies in these cities and residents’ policy adherence may account for the subtle differences.

# **3. App categorization**

Our dataset captured use logs for 14,000 smartphone apps. Most of them were used by a few users for a very short period. For the sake of interpretation, we narrowed the research scope by filtering apps according to the level of activity. We defined the activeness of an app by a minimum number of daily active users (DAU). An app is active in a given city if its DAU exceeds 1/5000 active smartphone users. Six hundred and seventy-nine apps were filtered out. We recruited three research assistants (RAs) to independently identify apps that provide similar services. RAs summarized 167, 210, and 180 groups of apps, respectively. The pairwise adjusted Rand Indices of the three groupings were 0.78, 0.75, and 0.73. Finally, the authors and RAs further adjusted the groupings. Fifty categories of apps were identified (**Table** S2).

**Table S2 App categorization**

| **App category** | **Description** | **Top DAU app** |
| --- | --- | --- |
| Mobility: navigation | For navigating within a city, hiring taxis, and sharing rides | 高德地图 |
| Mobility: travel | For booking flight tickets, railway tickets, and hotels for long-haul travel | 携程旅行 |
| Commercial: delayed delivery shopping | Online marketplace, in which the purchase will be delivered several days later | 拼多多 |
| Commercial: instant delivery shopping | Online marketplace, in which the purchase will be delivered immediately (usually within the day) | 叮咚买菜 |
| Commercial: O2O shopping | O2O stands for online-to-offline. For locating shops, receiving coupons, and making payments | 美团 |
| Commercial: discount offers | Apps that provide comparisons and discounts and redirect users to online marketplaces | 点淘 |
| Commercial: car sales | For comparing cars and receiving discounts | 懂车帝 |
| Commercial: payment | For making payments | 支付宝 |
| Commercial: bookkeeping | For keeping track of expenditures | 鲨鱼记账 |
| Commercial: seller backend | Used for running online shops | 微店 |
| Commercial: logistics | Logistic services for hire | 美团众包 |
| Work: productivity | Apps that can help with desk work, taking notes, or making drawings | WPS Office |
| Work: online collaboration | For collaborative work, e.g., online meetings | 钉钉 |
| Work: job hunting | For finding jobs | BOSS直聘 |
| Information: web browser | Browser software | 浏览器 |
| Information: news | Apps that provide real-time information | 今日头条 |
| WeChat | WeChat is an instant messenger but also provides all kinds of services | 微信 |
| Social: social network service (SNS) | Major online communities facilitating user-generated content and information sharing | 微博 |
| Social: dating | Chatting for finding casual romantic relationships | MOMO陌陌 |
| Social: matchmaking | Chatting for finding future spouses | 伊对 |
| Social: interest groups | For information sharing and chatting between strangers | LOFTER |
| Entertainment: short video | Short video platforms | 抖音(TikTok) |
| Entertainment: long video | Video platforms that purchased licenses for streaming TV shows and films | 腾讯视频 |
| Entertainment: live show | Live streaming shows hosted by ordinary people | 虎牙直播 |
| Entertainment: video downloading | Tools used for downloading files from the Internet, usually video | 百度网盘 |
| Entertainment: stimulating games | Games that attract people’s full attention | 王者荣耀 |
| Entertainment: casual games | Casual games | 开心消消乐 |
| Entertainment: gaming supports | Support services for gaming, such as game downloading and teammate hiring | 游戏中心 |
| Entertainment: music | Music streaming | 酷狗音乐 |
| Entertainment: audiobooks | Books that are read out by people or AI | 喜马拉雅 |
| Entertainment: karaoke | Apps that provide background music for people to sing along | 全民K歌 |
| Entertainment: novels | Novels and other light reading | 番茄免费小说 |
| Entertainment: comics | Comics and anime | 快看 |
| Education: under 12 (K12) | Education for kids under the age of 12 | 儿歌多多 |
| Education: under 18 (K18) | Education for adolescents under the age of 18 | 作业帮 |
| Education: adults | Education for adults | 下厨房 |
| Education: driving test | Education for driving test | 驾校一点通 |
| Living: local services | For getting local information and services | 58同城 |
| Living: house hunting | For finding housing, to purchase or to rent | 安居客 |
| Living: car services | Apps that provide car services | 平安好车主 |
| Living: exercising | Apps that track exercise | 健康 |
| Living: health | Apps that keep track of personal health or seek online medical resources | 美柚 |
| Living: new parents | Apps that track pregnancy and infant activity | 亲宝宝 |
| Living: finance | Apps that provide banking and loan services | 招商银行 |
| Living: investing | Apps that provide investment instruments, such as stocks and publicly offered funds | 京东金融 |
| Tools: translation | Language dictionary and translation | 网易有道词典 |
| Tools: image editing | For editing images and videos | 美图秀秀 |
| Tools: calendar | Calendars and schedules | 中华万年历 |
| Tools: weather | For checking the weather and air quality | 墨迹天气 |
| Tools: phone tools | WiFi connection and password management | 小布助手 |

# **4. Motivations behind the smartphone apps usage**

Theory suggests that the use of smartphone apps is driven by several internal factors, such as perceived usefulness and ease of use, as well as external factors, such as peer influence (Hew et al., 2015; Hsiao, Chang, & Tang, 2016). We assumed that in a sudden event, such as a city-level lockdown, only perceived usefulness — that is, the degree to which users believe that an app can fulfill their needs — would change, whereas all other factors would remain unchanged.

Maslow’s hierarchy categorizes needs into several levels along the material-to-intangible continuum: 1) biological and physiological needs, 2) safety, 3) love and belonging, 4) esteem, 5) cognitive, 6) aesthetic, and 7) self-actualization (Maslow, 1970). Inglehart (1977) further extended belonging and esteem to the realm of postmaterialism. Across 50 categories of smartphone apps, working and commercial groups fit into the lower physiological end of the need spectrum; information and social groups fit into the middle range where safety and social needs reside; education groups sit at the higher end of self-esteem and actualization. Both Maslow (1943, 1962, 1987) and Inglehart (1977) posited that an individual must fulfill needs at the lower end before progressing to higher levels.

Gratification theory suggests that audiences consume certain media for entertainment (Luo, & Remus, 2014). Various mobile apps, such as those involving gaming and music appreciation, provide people with a variety of leisure and entertainment activities. We adopted this view and added entertainment needs to the hierarchy of needs. We placed entertainment needs between safety needs and social needs. We hold that the need for entertainment is higher than safety needs but lower than social relationships and external recognition on the material-to-intangible or biological-to-spiritual ladder.

# **5. User behavior clustering**


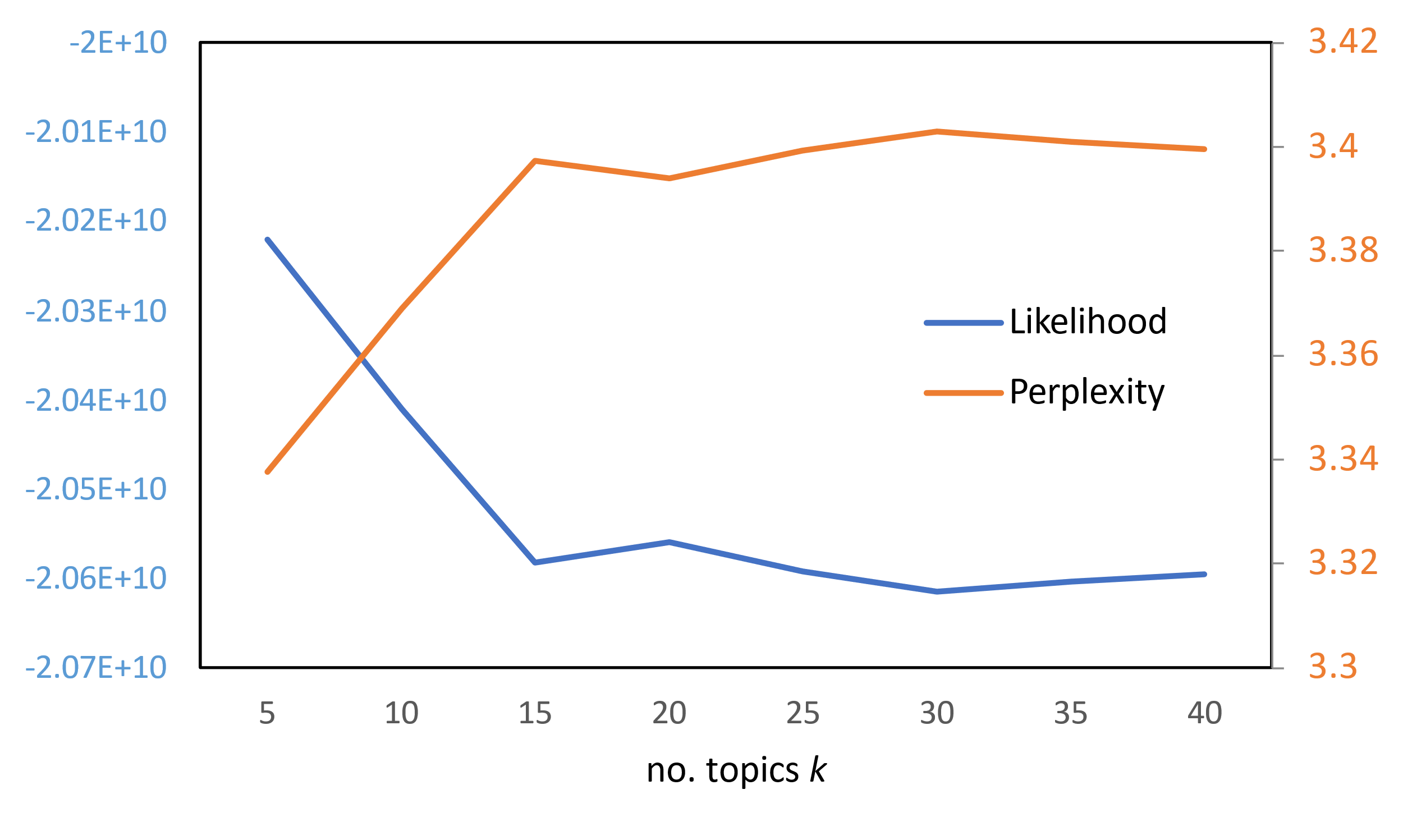


Figure S3 LDA’s likelihood and perplexity under different cluster numbers.

We used the latent Dirichlet allocation (LDA) algorithm to cluster users’ daily behaviors $\boldsymbol{p}_{u,d}$ in the entire observation periods. We used Spark’s parallel LDA algorithm with settings *maxIterations = 50, docConcentration = -1.0, topicConcentration = -1.0, seed = 36, checkpointInterval = 10,* and *optimizer = "online".* We tried different cluster sizes *k* from five to forty, with an interval of five. Twenty clusters yielded the best likelihood and perplexity (**Fig.** S3).

User clustering is analogous to topic modeling in natural language processing. Each cluster can be interpreted using signature apps. The signature apps of the 20 clusters are shown in **Table** S3. All clusters exhibited excessive use of WeChat^[[2]](#footnote-2)^, short videos, and delayed delivery shopping apps. All but one cluster displayed one or two other app categories as their signature. The cluster with no other signature app categories is also the largest (19\% of the population). Consequently, we refer to that cluster as regular users.

We also tried to interpret clusters with *k* = 15 and 25. With 15 clusters, the most commonly used apps were again WeChat, short video, and delayed delivery shopping apps. However, the signature apps of most clusters were not apparent, i.e., they did not have the highest weights. Therefore, we considered that 15 clusters might not differentiate user behavior patterns. With 25 clusters, more sub-clusters appeared, with gaming apps serving as the signature. We do not think these clusters can be interpreted with clearly distinct meanings. Eventually, we chose 20 clusters.

Table S3 Signature apps of the 20 user clusters with weights given by the LDA algorithm.

| **User cluster** | **% population** | **Signature apps (weights)** | | | |
| --- | --- | --- | --- | --- | --- |
| Regular users | 19% | WeChat (0.30) | Entertainment: short video (0.28) | Commercial: delayed delivery shopping (0.15) | Information: web browser (0.07) |
| Binge-watchers | 9% | **Entertainment: long video (0.20)** | WeChat (0.18) | Entertainment: short video (0.14) | Commercial: delayed delivery shopping (0.11) |
| Gamers | 9% | **Entertainment: stimulating games (0.20)** | Entertainment: short video (0.16) | WeChat (0.15) | Entertainment: music (0.12) |
| Telecommuters | 8% | WeChat (0.15) | Entertainment: casual games (0.15) | **Work: online collaboration**  **(0.13)** | Entertainment: short video (0.13) |
| News audience | 8% | **Information: news (0.26)** | WeChat (0.18) | Entertainment: short video (0.12) | Living: local services (0.10) |
| Drivers | 6% | WeChat (0.17) | **Mobility: navigation (0.16)** | Entertainment: short video (0.14) | Commercial: payment (0.13) |
| Photoshoppers | 5% | **Tools: image editing (0.13)** | WeChat (0.13) | Entertainment: short video (0.10) | Commercial: delayed delivery shopping (0.10) |
| Spenders | 4% | **Commercial: online-to-offline (O2O) (0.19)** | WeChat (0.11) | Entertainment: short video (0.09) | **Commercial: cars purchase (0.08)** |
| Adolescents & young adults | 4% | **Education: under 18 (K18) (0.19)** | WeChat (0.13) | Entertainment: short video (0.10) | **Living: house hunting (0.09)** |
| Travelers | 4% | **Entertainment: novels (0.22)** | **Mobility: travel (0.15)** | WeChat (0.13) | Commercial: delayed delivery shopping (0.08) |
| Petty investors | 3% | **Living: finance (0.32)** | WeChat (0.13) | Entertainment: short video (0.10) | Commercial: payment (0.07) |
| Homemakers | 3% | **Tools (weather) (0.30)** | **Commercial: bookkeeping (0.18)** | WeChat (0.11) | Entertainment: short video (0.10) |
| White collars | 3% | **Work: productivity (0.18)** | Entertainment: live show (0.15) | WeChat (0.11) | Information: web browser (0.09) |
| Love hunters | 3% | WeChat (0.13) | **Social: dating (0.11)** | Entertainment: music (0.10) | Entertainment: short video (0.09) |
| Sophisticated users | 3% | **Information: web browser (0.17)** | WeChat (0.16) | **Entertainment: video downloading (0.15)** | Commercial: delayed delivery shopping (0.09) |
| Job hunters | 2% | WeChat (0.11) | **Work: job hunting (0.11)** | Living: investing (0.10) | Information: web browser (0.09) |
| New parents | 2% | **Living: new parents (0.21)** | Education: adults (0.16) | Social: matchmaking (0.10) | WeChat (0.09) |
| New middle class | 2% | Tools: calendar (0.23) | **Education: driving test (0.13)** | WeChat (0.10) | Social: interest groups (0.10) |
| Parents with small children | 2% | Living: health (0.24) | **Education: under 12 (K12) (0.19)** | WeChat (0.11) | Entertainment: short video (0.09) |
| Home shoppers | 2% | **Commercial: instant delivery shopping (0.19)** | WeChat (0.13) | **Commercial: discount (0.10)** | Commercial: delayed delivery shopping (0.09) |

# **6. Usage time for each app category and the DDID effects**

Each app category’s absolute daily use time in the lockdown and control cities and the DDID effects are shown in **Fig.** S4 and S5. DDID effects of relative daily app use is shown in **Fig.** S6. Lockdowns have larger or contradicting effects to some apps in some cities (especially Shijiazhuang and Nanjing). Therefore, an ablation study was further conducted. **Figs.** S7 and S8 plot the DDID effects of relative daily app use (similar to **Fig.** S6), excluding Shijiazhuang and Nanjing, respectively. Effect heterogeneity can be found in some app categories. For example, the overall effects of “instant delivery shopping” in the second fortnight, “productivity” in the second fortnight and after the lockdown, “gaming supports” during the lockdown, “comics” during the lockdown, “interest groups” in the first fortnight, and “image editing” during the lockdown are changed when excluding Shijiazhuang. “Delayed delivery shopping” did not show significant change. Meanwhile, “payment” during the lockdown and “interest groups” after the lockdown are also changed when excluding Nanjing from the pooled sample. However, as the directions and significance of most effects are preserved, we argue that the main conclusion of this paper is not affected by the effect heterogeneity.


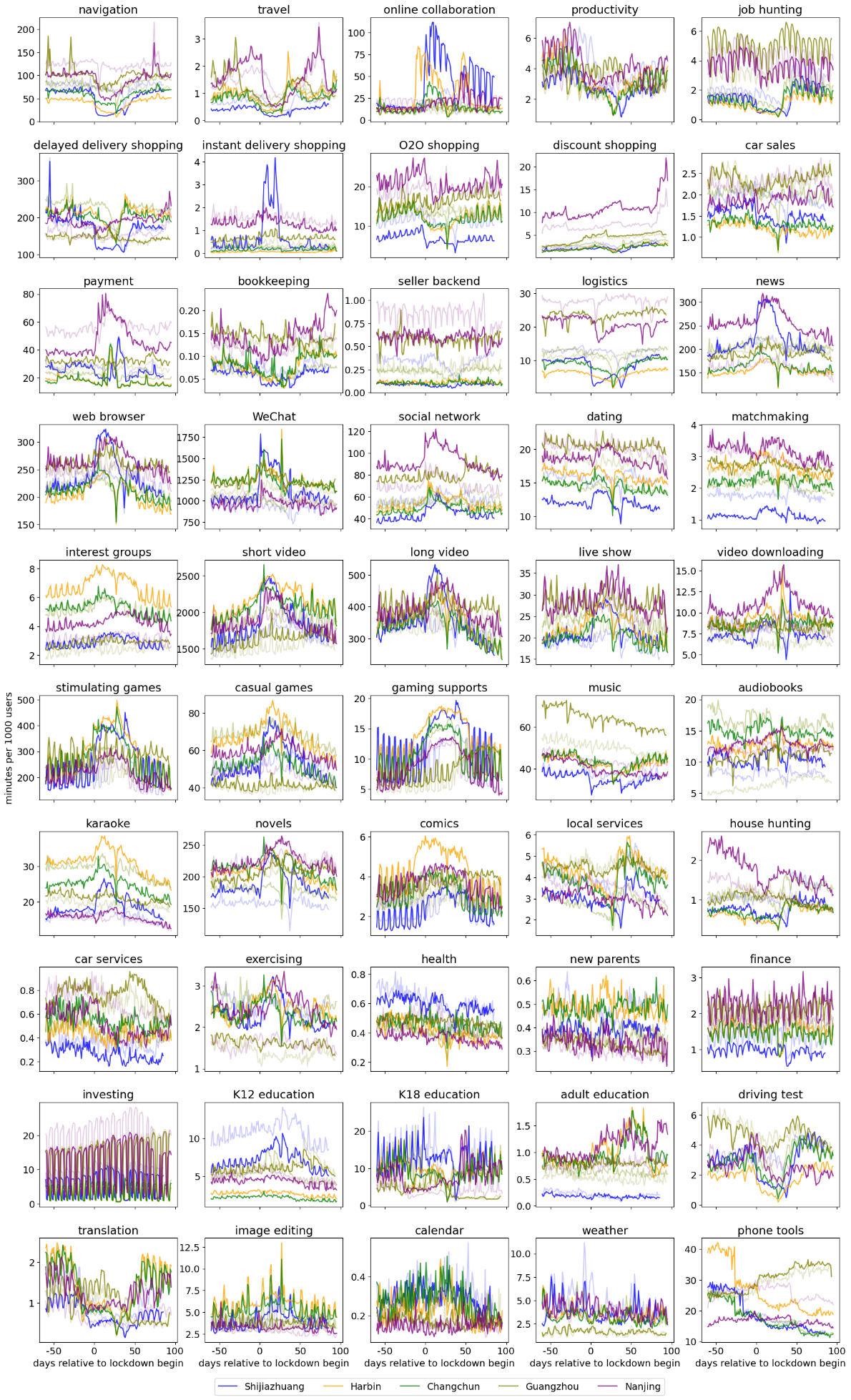


Figure S4 Absolute use time for 50 app categories. Opaque lines represent use time in lockdown cities, and transparent lines represent corresponding control cities.


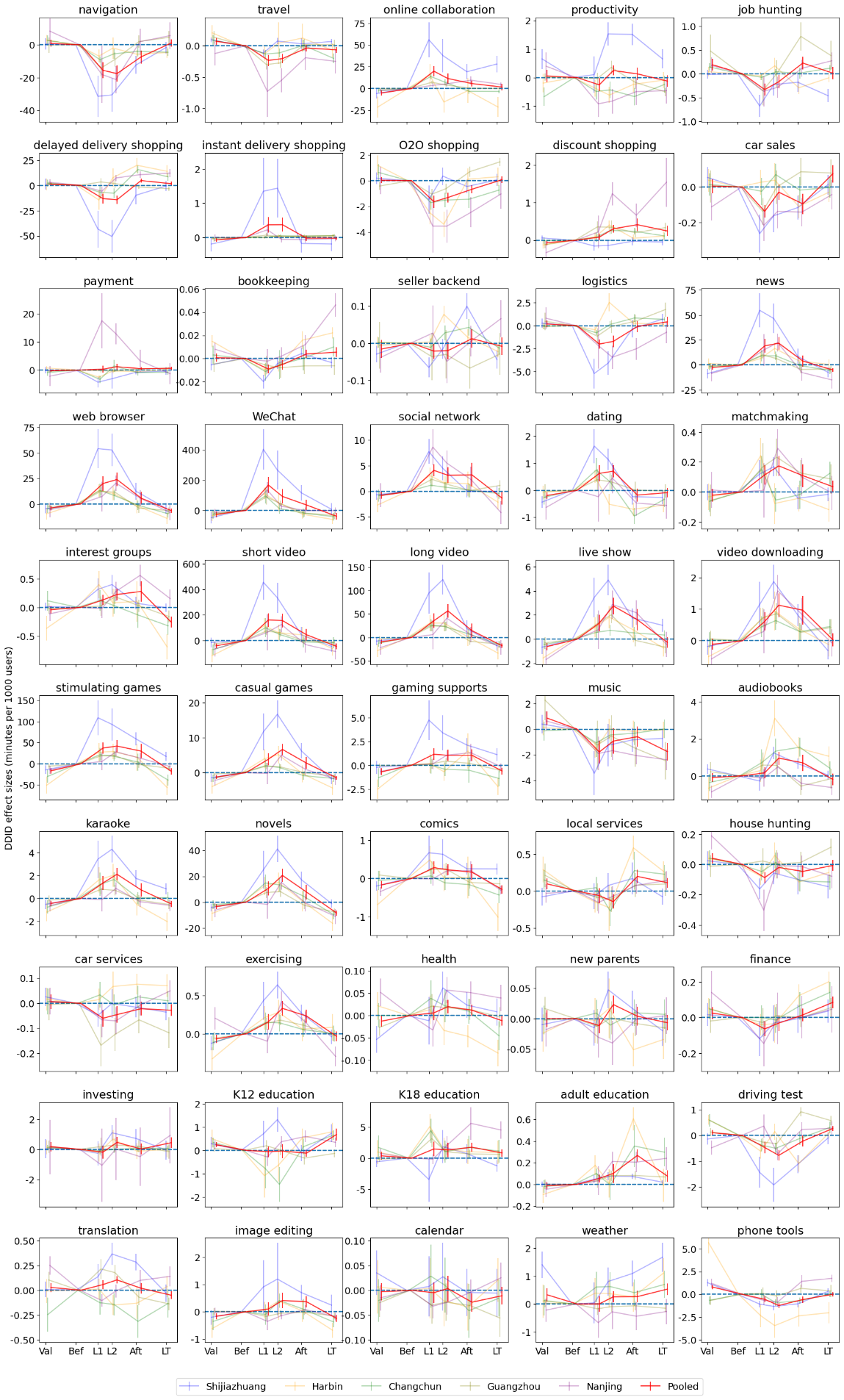


Figure S5 DDID effects of absolute app use time. On the x-axis: Val: 30-60 days before lockdown, Bef: the month before lockdown, L1 and L2: the first and second fortnights after lockdown, Aft: one month after lockdown, and LT: the second month after lockdown.


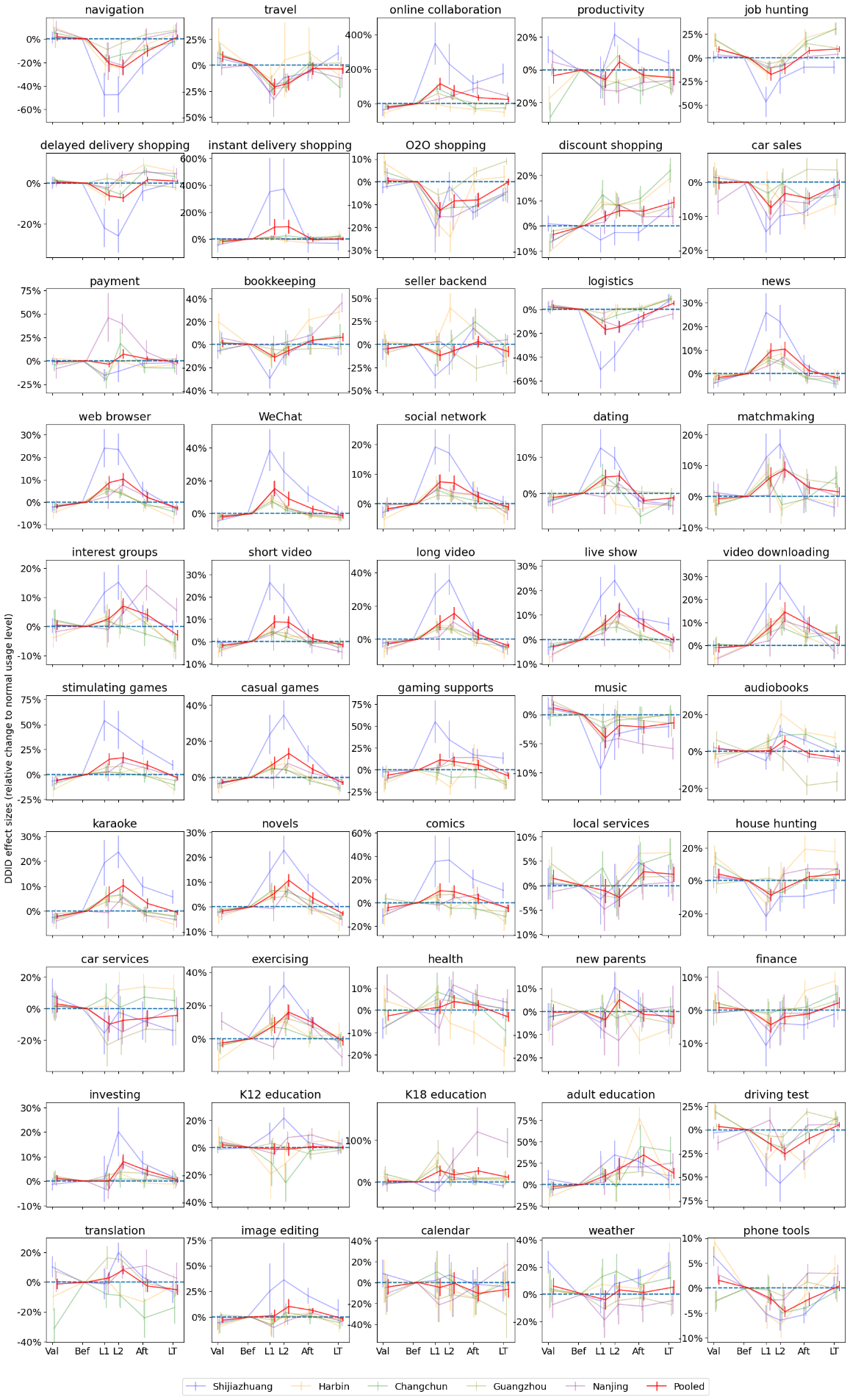


Figure S6 DDID effects of relative app use time. On the x-axis: Val: 30-60 days before lockdown, Bef: the month before lockdown, L1 and L2: the first and second fortnights after lockdown, Aft: one month after lockdown, and LT: the second month after lockdown.


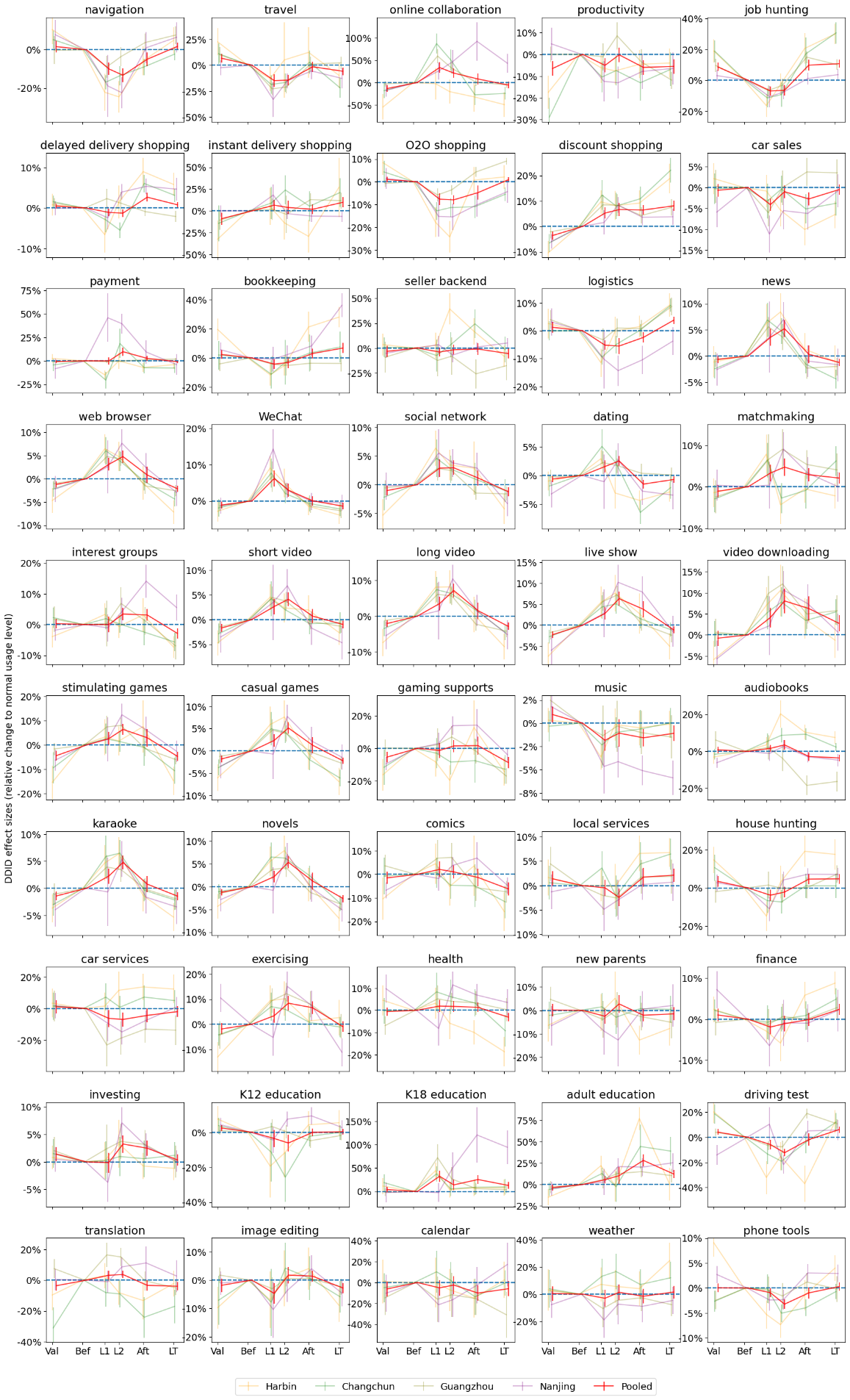


**Figure S7 DDID effects of relative app use time with Shijiazhuang excluded.** On the x-axis: **Val**: 30-60 days before lockdown, **Bef**: the month before lockdown, **L1** and **L2**: the first and second fortnights after lockdown, **Aft**: one month after lockdown, and **LT**: the second month after lockdown.


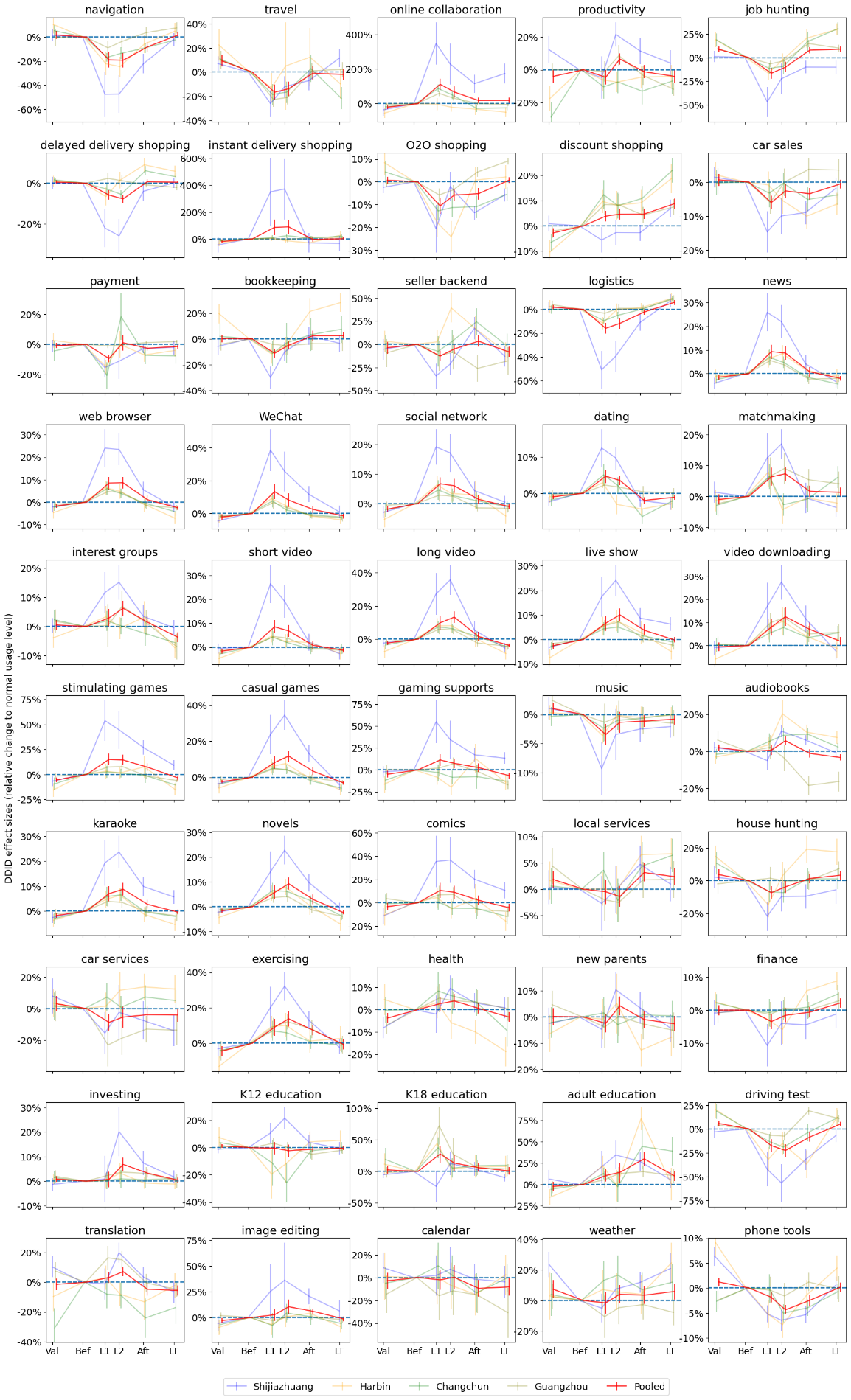


**Figure S8 DDID effects of relative app use time with Nanjing excluded.** On the x-axis: **Val**: 30-60 days before lockdown, **Bef**: the month before lockdown, **L1** and **L2**: the first and second fortnights after lockdown, **Aft**: one month after lockdown, and **LT**: the second month after lockdown.

# **7. Change of user cluster sizes and the DDID effects**

The sizes of clusters over time in each city are shown in **Fig.** S9. The DDID effects of cluster size measured by the proportion in the population changes are shown in **Fig.** S10. **Fig.** S11 shows the change of each cluster relative to the period before the lockdown.

To characterize lockdowns’ disruptions to routine behaviors, we looked at the number of users who transited from one cluster to another during a given time window. Transitions among user clusters form a matrix $\boldsymbol{A}=a_{ij},i,j\in\left\{ 1,\ldots,20 \right\}$, where $a_{ij}$ is the number of people who moved from cluster $i$ to $j$ minus those who moved from cluster $j$ to $i$. Smartphone use data has a clear weekly period (see **Figs.** S2, S4, and S9), so we set the window size to seven days. Similar to the definition of lockdown start dates, we considered the impact of lockdowns on user behavior change as the window with the strongest fluctuation of the transition matrix measured in standard deviation. For Shijiazhuang, Harbin, Changchun, Guangzhou, and Nanjing, the window start dates were six, four, four, six, and one day ahead of lockdown, respectively (see **Fig.** S12).


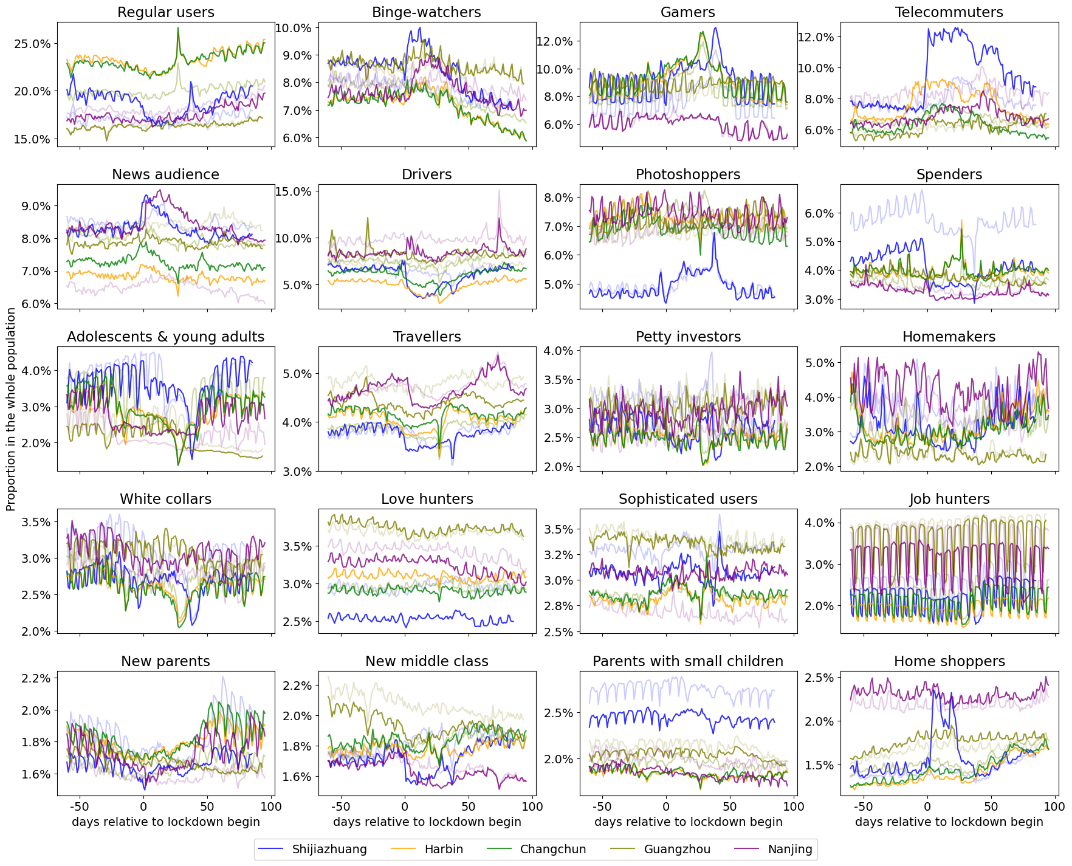


Figure S9 Cluster sizes over time in each city. Solid lines represent cluster sizes (proportion of the whole population) in lockdown cities. Transparent lines represent control cities.


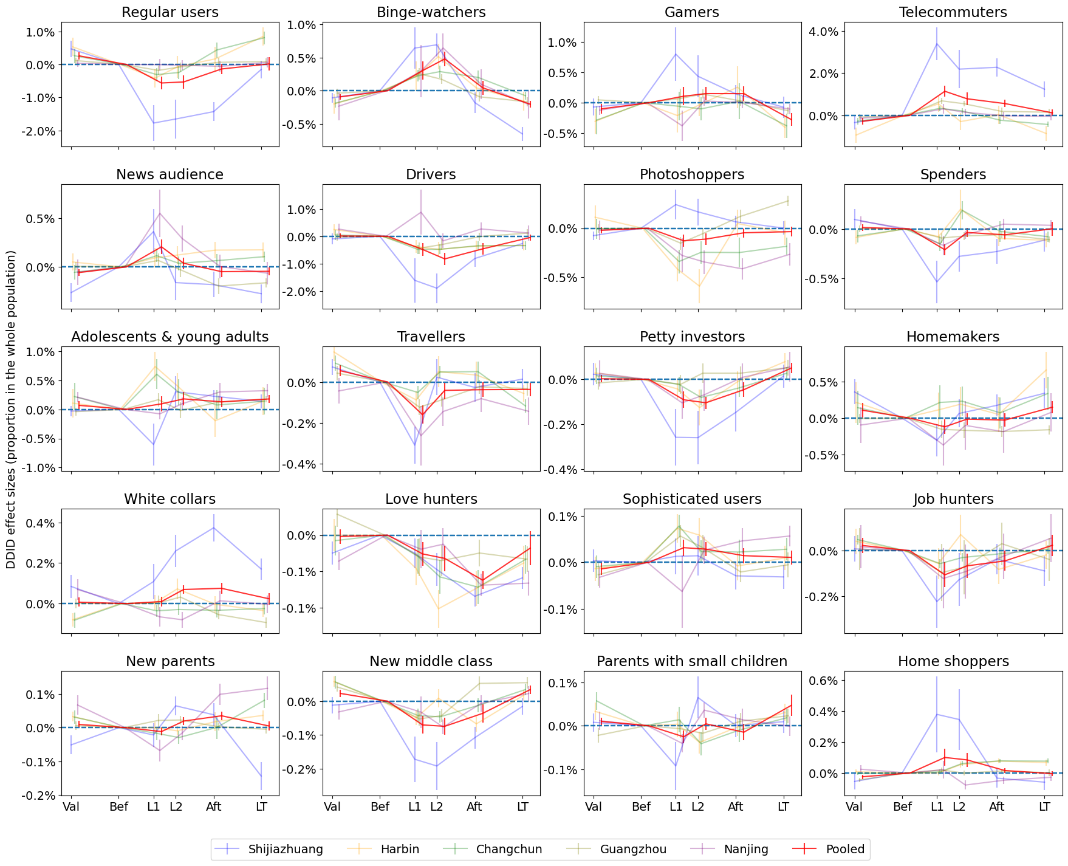


Figure S10 DDID effect sizes of changes in user cluster size (by the proportion in the population). On the x-axis: Val: 30-60 days before lockdown, Bef: the month before lockdown, L1 and L2: the first and second fortnights after lockdowns began, Aft: one month after lockdown, and LT: the second month after lockdown.


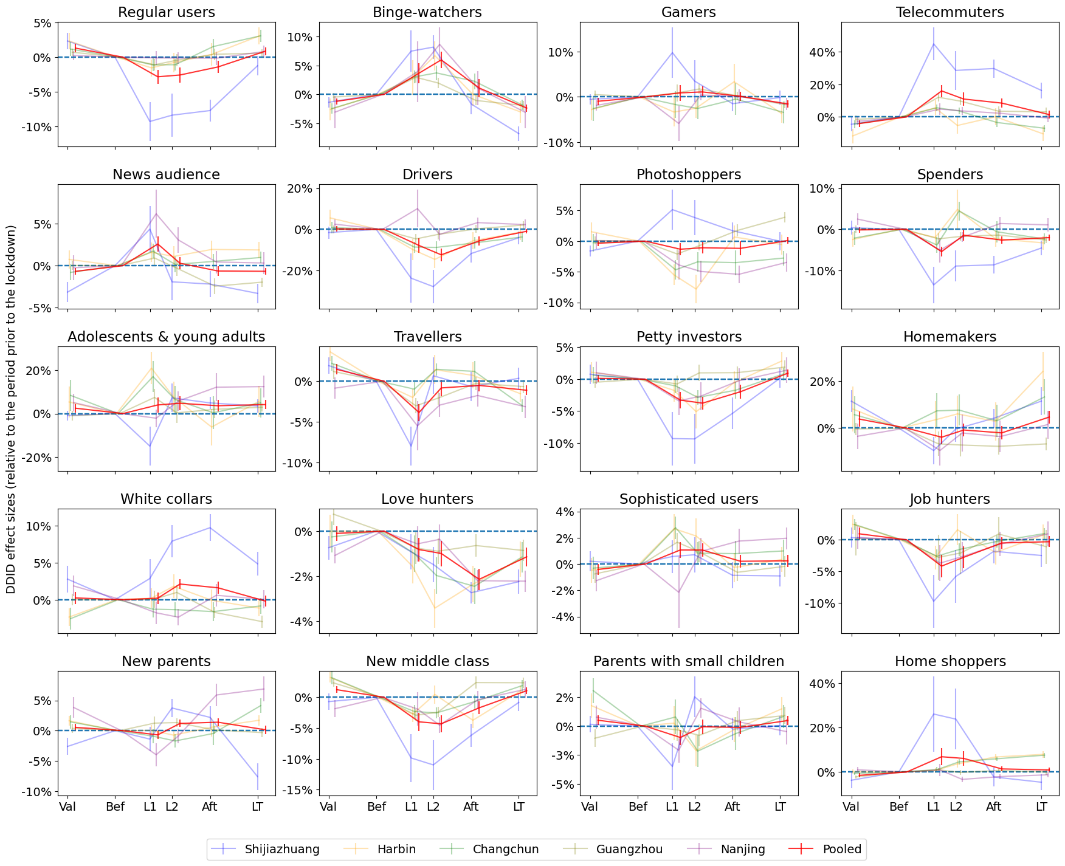


Figure S11 DDID effect sizes of changes in user cluster size (relative to the period prior to the lockdown). On the x-axis: Val: 30-60 days before lockdown, Bef: the month before lockdown, L1 and L2: the first and second fortnights after lockdowns began, Aft: one month after lockdown, and LT: the second month after lockdown.


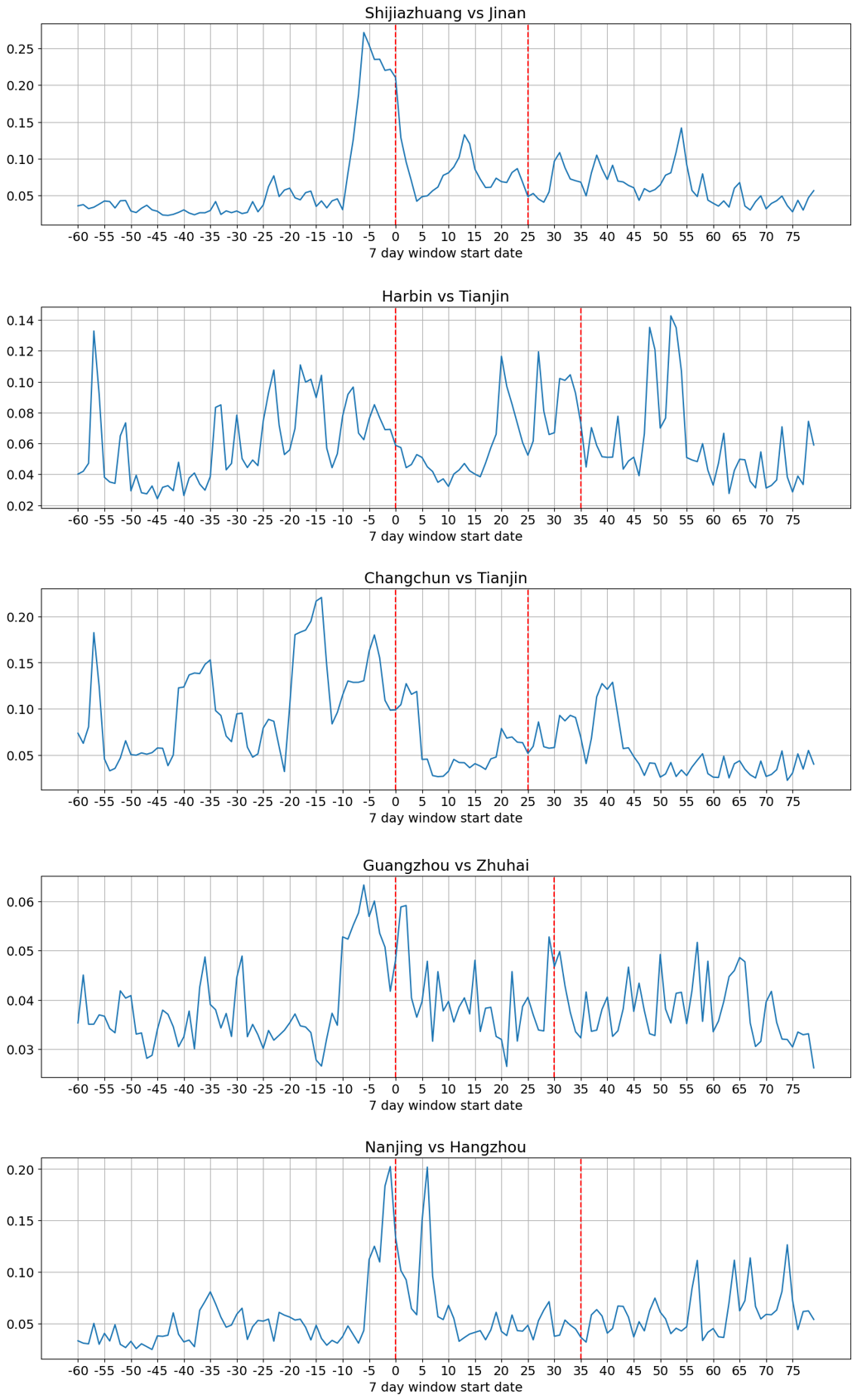


Figure S12 Selection of behavior transition window start dates. Blue lines are the standard deviation of the transition matrices with window size 7. Dashed lines are lockdown start and end dates.

# **8. The User Experience Improvement Program Privacy Statement**

User Experience Program

Last updated: August 23, 2021

Welcome to the User Experience Program!

The User Experience Program (hereinafter referred to as the “Program”) is designed to improve the user experience of the product by continuously improving product performance and introducing services that are helpful to you. To achieve the purpose mentioned above, during the Program, we may need to collect information about your device (which may include your personal information). This Privacy Statement will help you understand the purpose, method and scope of our collection of your personal information, and how we use and protect your personal information.

If you disagree with any provision in the Privacy Statement, you may tap “Disagree” to withdraw from the Program. By tapping “Agree” to enable the Program, you fully understand how information is collected and used as described in the following sections of this Privacy Statement, as well as matters such as those relating to your rights, and you agree that we can collect and use the information you provide to us and information about your device.

I. How We Collect and Use Your Personal Information

You agree to our collection of the following data from your device for our analysis. Meanwhile, this Program requires a network connection, and some data can only be uploaded over Wi-Fi:

• Your device information, such as device data, configuration information, system and application data, advertising ID, IP address, phone model and firmware version information, system debugging status, ROM and RAM status, clock information, device identification numbers, device operating environment information, and hardware information (including display, CPU, sensor, and battery).

• Your operational behavior data, such as product function usage statistics, product upgrade statistics, as well as data about app installation, uninstallation, error logs, etc.;

• If you allow apps to track location, we may receive some information about the location of the user device;

• Your log information, such as the time at which the service is utilized and the duration of use, search words that have been entered using the service, and information about device and software events (such as reboots, upgrades, errors, crashes, etc.).

• App/SDK information, such as app package name, app version, app name, app signature, app size, and app or SDK version number.

• Network information, such as SIM card status, network type, base station information, network information, and virtual private network information.

After collecting your relevant information, we may perform statistical analysis on it and use the aggregated analysis results to:

• Improve the functional experience of the product and determine the quality of channels;

• Gather statistics on the use of various product functions and analyze the statistical data to continuously improve the product in terms of user-friendliness, operational performance, functions and design;

• Analyze your behaviors and preferences to send you information about our services or other products and services that we think you may be interested in, which include but are not limited to product services and personalized ads;

• Detect risks to devices to generate security protection policies and launch corresponding security features.

• Count the number of users for product marketing and promotion;

• Analyze error logs to improve phone quality and app functions;

• Implement other applications based on the statistical results.

We will strictly abide by the purposes stated in the aforementioned when your personal information is used. Your personal information will only be used for the purposes that have been established at the time of collection with your consent. In the event that such information may be used for any other purposes, we will seek your prior consent before we proceed. We keep strictly confidential all the information collected from your device by taking various security measures when processing the data, and implementing security measures like storage and transmission encryption, so as to protect your information from unauthorized access, use or disclosure, and to ensure that the information is transferred securely.

We only store your information for the minimum amount of time required. Once we complete the statistical analysis of this Program, we will delete or anonymize all the data collected from your device for the Program.

II. How We Disclose Your Personal Information

To accomplish the above goals of analysis, we may share some collected information with the authorized partners. For example, we will provide the records regarding your downloading and uninstalling apps to “App Market” for it to recommend to you the products you may take an interest in. We will only share your personal information for purposes that are lawful, justifiable, necessary, specific and definite, and will only share such personal information on an as-needed basis for service provision. Unless otherwise stated in this Privacy Statement, we will not disclose your personal information to third parties without your prior consent for their respective independent marketing or commercial use.

We will require the third parties to take appropriate confidentiality and security measures to process personal information through agreements or other appropriate measures.

III. Miscellaneous

You can opt out of this Program at any time in “Settings - About this phone – Experience improvement programs”. After you opt out of this Program, all data collected from your device for the purposes of the Program will be deleted or anonymized.

We may modify this privacy statement and issue relevant information in a conspicuous location for the products in order to timely notify you hereof.

Regarding the processing of the personal information involved in this Program, for information on any specific privacy policy issues not dealt with in this Privacy Statement (including but not limited to “How We Retain Your Personal Information”, “Your Rights to Your Personal Information”, “How We Handle Personal Information about Children”, “Third-party Service Providers and Their Services”, and “How Your Personal Information Is Transferred Globally”), see our Privacy Notice. To view the details of the Privacy Notice, go to “Settings - About phone - Legal information – Privacy Notice”.

# **References**

Hew, J. J., Lee, V. H., Ooi, K. B., & Wei, J. (2015). What catalyses mobile apps usage intention: an empirical analysis. *Industrial Management & Data Systems, 115*(7).

Holtz, D., Zhao, M., Benzell, S. G., Cao, C. Y., Rahimian, M. A., Yang, J., Allen, J., Collis, A., Moehring, A., Sowrirajan, T., Ghosh, D., Zhang, Y., Dhillon, P.S., Nicolaides, C., Eckles, D., & Aral, S. (2020). Interdependence and the cost of uncoordinated responses to COVID-19. *Proceedings of the National Academy of Sciences*, *117*(33), 19837-19843. <https://doi.org/10.1073/pnas.2009522117>

Hsiao, C. H., Chang, J. J., & Tang, K. Y. (2016). Exploring the influential factors in continuance usage of mobile social Apps: Satisfaction, habit, and customer value perspectives. *Telematics and Informatics*, *33*(2), 342-355. <https://doi.org/10.1016/j.tele.2015.08.014>

Inglehart, R. (1977). *The Silent Revolution: Changing Values and Political Styles Among Western Publics*. Princeton University Press.

Killick, R., Fearnhead, P., & Eckley, I. A. (2012). Optimal detection of changepoints with a linear computational cost. *Journal of the American Statistical Association*, *107*(500), 1590-1598. <https://doi.org/10.1080/01621459.2012.737745>

Luo, M. M., & Remus, W. (2014). Uses and gratifications and acceptance of Web-based information services: An integrated model. *Computers in Human Behavior*, *38*, 281-295. <https://doi.org/10.1016/j.chb.2014.05.042>

Maslow, A. H. (1970). *Motivation and personality*. Harper & Row.

Maslow, A. H. (1943). A theory of human motivation. *Psychological Review,* *50*(4), 370–396.

Maslow, A. H. (1962). *Toward a psychology of being*. D. Van Nostrand Company.

Maslow, A. H (1987). *Motivation and personality (3rd ed.)*. Pearson Education.

1. <https://huiyan.baidu.com/> [↑](#footnote-ref-1)
2. WeChat originated as an instant messenger app in 2011 and developed into an all-in-one app that supports many functionalities by allowing applets to run inside the main app. [↑](#footnote-ref-2)
